# Cryoablation for Renal Cell Carcinoma Prior to Liver Transplantation: A Case Report

**DOI:** 10.7759/cureus.32531

**Published:** 2022-12-14

**Authors:** Neal Modi, Kristen Dougherty, Mustafa Nazzal

**Affiliations:** 1 Department of Surgery, Saint Louis University School of Medicine, St. Louis, USA; 2 Department of Surgery, Saint Louis University Hospital, St. Louis, USA

**Keywords:** disease-free survival, transplant hepatology, extrahepatic malignancy, hepatocellular carcinoma (hcc), renal carcinoma recurrence, nephrectomy, pre-transplant evaluation, percutaneous cryoablation, orthotopic liver transplantation, renal cell carcinoma (rcc)

## Abstract

Extrahepatic malignancies are a relatively rare incidental finding during liver transplant work-up that provides a significant barrier to continued transplant evaluation and requires treatment to limit the risk of recurrence. There have only been 11 previously reported cases of pre-liver transplant renal cell carcinoma (RCC), of which all underwent partial or radical nephrectomy. Percutaneous cryoablation therapy has been gaining acceptance as a curative treatment alternative for RCC and is a new therapeutic standard for patients who are poor candidates for surgical resection. Recent studies have demonstrated the safety and efficacy of cryoablation for RCC in native kidneys and in solid masses in kidney allografts, but there is no data on the efficacy or recurrence of RCC when cryoablation is used for the treatment of RCC in a native kidney prior to solid organ transplantation.

The patient underwent percutaneous cryoablation therapy of a T1a RCC of the native kidney 10 months prior to orthotopic liver transplant (OLT) without subsequent partial or radical nephrectomy. At seven years post-ablation therapy, the patient has no evidence of tumor recurrence despite immunosuppressive therapy post-transplantation.

Cryoablation is potentially a safe and highly effective means of treating RCC in patients who are not candidates for nephrectomy secondary to complications associated with end-stage liver disease. In our case, the patient was treated with cryoablation and received standard post-transplant immunosuppression without recurrence of RCC at seven years. More studies are needed to determine inclusion and exclusion criteria for cryoablation and to confirm long-term efficacy as well as a strategy for duration and frequency of surveillance in these patients.

## Introduction

Extrahepatic malignancies are a relatively rare incidental finding during liver transplant work-up. Four large retrospective studies identified only 1.6% to 6.1% of patients who had preexisting extrahepatic malignancy prior to orthotopic liver transplant (OLT) [1-4]. Furthermore, only 11 previous cases of pre-liver transplant renal cell carcinoma (RCC) have been reported, of which six were discovered prior to the transplantation process and five were discovered during liver transplant work-up and/or at the time of liver transplantation [1-6]. RCC identified during work-up of a solid organ transplant candidate requires treatment to limit the risk of recurrence [7]. The American Urological Association (AUA) provides guidelines on the management of small renal masses (SRMs) suspicious of RCC. Mainstay treatment options for RCC include radical nephrectomy or partial nephrectomy. The AUA offers a moderate recommendation to prioritize partial nephrectomy for T1a renal masses and also provides guidance on the use of thermal ablation as a standard alternative approach for SRMs less than three centimeters [8]. Occasionally in SRMs, a watchful approach could be utilized; however, for those awaiting transplantation, nephrectomy remains the standard approach of SRM treatment [9,10].

Of the 11 reported cases, eight patients underwent radical nephrectomy (before, during, or within three months following OLT), one patient underwent partial nephrectomy, and two patients died of complications shortly after OLT (RCC discovered in OR). None underwent non-surgical treatment options for renal masses including thermal ablation, cryoablation, and radiofrequency ablation [11]. After appropriate treatment, T1a RCCs (< 4 cm) have a 95-98% recurrence-free survival rate after five years. There is currently no recommended minimum surveillance period between treatment of these tumors and solid organ transplantation, likely because the three-year probability of metastasis is less than 2% [7,11].

Prolonged immunosuppression post-transplantation is a major contributor to the elevated risk of de novo and recurrent malignancies in liver transplant recipients due to the immune system’s diminished ability to prevent malignant transformation [3]. As such, extrahepatic malignancies in the pre-transplantation period are generally considered a contraindication to liver transplant due to unacceptable risk of recurrence [6]. Liver transplant patients pose an even more unique challenge to therapeutic management of renal neoplasms as they are often poor surgical candidates for elective abdominal procedures due to complex portosystemic venous communications in the retroperitoneum and other complications associated with portal hypertension [10]. These sequelae of cirrhosis create a prohibitively high peri-operative mortality for most liver transplant candidates.

Percutaneous cryoablation therapy has been gaining acceptance as a curative treatment alternative to surgery for T1a RCC and is a therapeutic standard for patients who are poor candidates for resection or those who prefer to avoid surgical intervention [12]. Prospective studies have demonstrated the safety and efficacy of minimally invasive nephron-sparing cryoablation for T1a/T1b RCCs with an overall five-year survival rate of 97.8%, comparable to that of partial nephrectomy [13]. There is some evidence to suggest potential efficacy of percutaneous cryoablation of RCC in transplanted kidneys [14]. A systematic review of 163 patients with solid masses in kidney allografts (88% of which were found to be RCCs) found that of the four patients (2.4%) who underwent percutaneous cryoablation therapy, none experienced local recurrence at a mean follow-up of 17 weeks [4,15]. However, there are no data or reports on the efficacy or recurrence rates of RCC when cryoablation is used for the treatment of RCC in a native kidney prior to solid organ transplantation.

We present the case of a patient with end-stage liver disease secondary to non-alcoholic steatohepatitis (NASH) cirrhosis and hepatocellular carcinoma (HCC), in which an RCC was incidentally discovered during pre-transplant work-up and treated with interventional radiology (IR) image-guided cryoablation therapy rather than conventionally accepted radical nephrectomy or partial nephrectomy due to unacceptably high peri-operative risk.

This is a case report with data collected from a retrospective chart review. The Saint Louis University Institutional Review Board (IRB) has determined that case reports including five or less subjects are exempt from needing IRB review.

## Case presentation

A 53-year-old male patient with a history significant for hypertension, type 2 diabetes mellitus, obesity, peptic ulcer disease, colon adenomas, and cholecystectomy was being followed by the hepatology service in 2013 for a three-year history of NASH cirrhosis. At the time, his model for end-stage liver disease (MELD) score was 9. His cirrhosis was complicated by large esophageal varices and portal hypertensive gastropathy. Routine multiphasic contrast computed tomography (CT) of the abdomen for HCC in April 2013 revealed a 5-mm enhancing exophytic mass within the lower pole of the right kidney, concerning for a small RCC. Other relevant CT findings included a 4-mm non-specific arterial enhancing focus within hepatic segment V with recommended continued surveillance and an enhancing focus within the pancreatic tail in the arterial phase suspicious for a small pseudoaneurysm.

The patient was subsequently referred to the urology service for management in October 2013. He was asymptomatic without urinary frequency, dysuria, flank pain, or gross hematuria, but had a history of microhematuria noted on previous urinalysis. He denied a family history of urologic problems, including renal malignancies. Flexible cystoscopy for characterization of microhematuria was unremarkable, and the decision was made to continue to monitor renal lesion on CT scans for continued HCC surveillance every six months.

Interval multiphasic contrast CT scan in March 2014 showed increase in size of the right lower pole exophytic renal lesion to 9 mm. This was not well visualized on follow-up retroperitoneal ultrasound, but a new large Bosniak 2F cyst was discovered at the inferior pole of the left kidney with thick septations.

**Figure 1:**
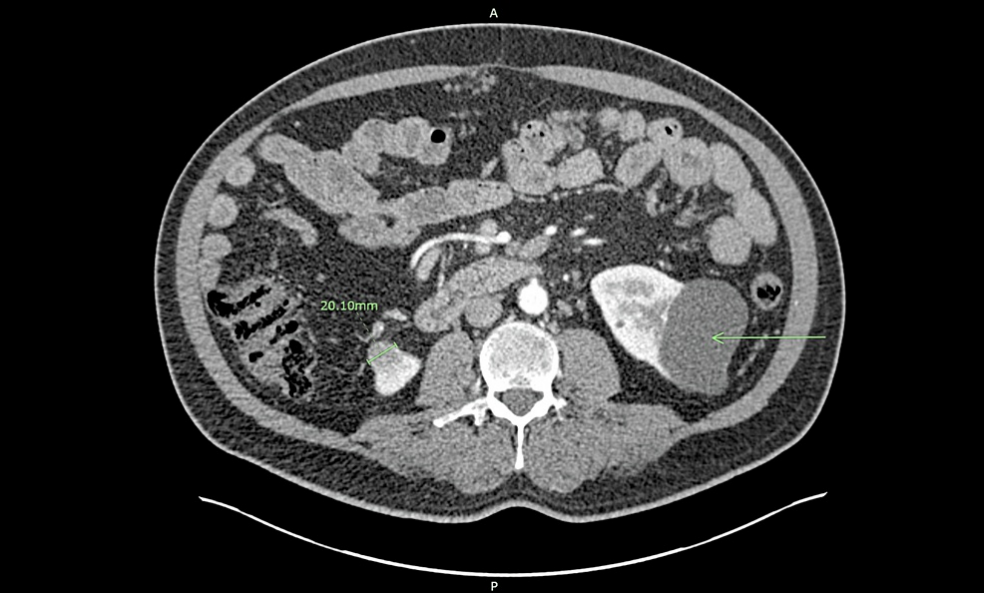
Axial view of the right renal inferior pole mass A right renal inferior pole mass measuring 20.10 mm can be visualized in this axial view along with a 70.86-mm left renal inferior pole lobulated cyst (arrow).

**Figure 2:**
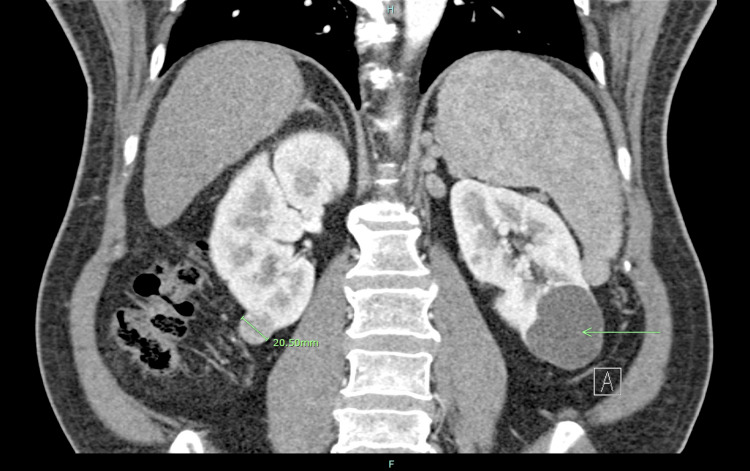
Coronal view of the right renal inferior pole mass A right renal inferior pole mass measuring 20.50 mm can be visualized in this coronal view along with a 52.58-mm left renal inferior pole lobulated cyst (arrow).

Routine six-month follow-up multiphasic contrast CT in November 2014 showed a further increase in the exophytic right inferior pole mass to 2 cm and an unchanged 7.0 cm lobulated cyst in the left renal lower pole (Figures 1, 2). There were also newly visualized cysts in the left and right kidneys and the development of a new 2-cm arterially enhancing Organ Procurement and Transplant Network (OPTN) 5A lesion with washout in hepatic segment VIII that was consistent with HCC.

At this time, the hepatology service referred the patient to initiate work-up for orthotopic liver with possible right partial nephrectomy at the time of liver transplantation. Meanwhile, referral was also made to IR service with a plan to treat the identified hepatic segment VIII HCC with trans-arterial chemoembolization (TACE) and to treat the suspected RCC on imaging with CT-guided cryoablation therapy; both procedures were successfully performed in January 2015. Pathology results from right renal mass biopsy revealed a low-grade clear cell RCC.

Follow-up magnetic resonance imaging (MRI) in March 2015 showed a complete response of the renal mass to cryoablation therapy with no residual enhancement or recurrent mass, complete response of hepatic segment VIII HCC to TACE, a new 1.1-cm hepatic segment IVa OPTN 4 lesion with washout, and two additional non-specific foci of arterial enhancement without washout.

After completing all liver transplant work-up, the patient was activated on the United Network for Organ Sharing (UNOS) recipient waiting list for liver transplant on March 16, 2015, with a MELD score of 10. Serial MRI studies throughout 2015 every two months were performed until October 2015 for post-intervention monitoring, which continued to demonstrate no significant interval changes in the post-TACE hepatic segment VIII lesion and the post-cryoablation right inferior renal pole lesion. Both were without evidence of residual or recurrent disease. All other identified hepatic lesions remained unchanged, and there were no other new hepatic or renal lesions discovered on MRI except for a small non-occlusive thrombus in the right and main portal veins.

In November 2015, the patient underwent OLT with end-to-end hepatic vein reconstruction with choledo-choledochostomy. During the procedure, after the inferior vena cava (IVC) was clamped and the recipient liver was removed, the urology team attempted to proceed with the planned right partial nephrectomy, but it was aborted due to the patient’s hemodynamic instability and unexpected extensive dissection requirement that would considerably increase the time the IVC remained clamped. Post-operatively, the patient was started on immunosuppression per our institution’s protocol with no deviations. No induction was given, and the patient received high-dose methylprednisolone taper and then transition to prednisone, mycophenolate mofetil, and tacrolimus.

Hepatic explant pathology revealed the treated HCC tumor site at hepatic segment VIII (1.2 cm) and multiple additional foci of well-differentiated HCC in segment I (1.2 cm), segment IVa (1.4 cm and 1.7 cm), segment VI (0.9 cm), and segment VII (0.9 cm). There was no evidence of lymph node metastasis. HCC was staged as pT2N0M0. His immediate post-transplant course was complicated by *Enterobacter aerogenes* and methicillin-sensitive *Staphylococcus aureus* pneumonia. He was discharged home on appropriate antibiotic therapy. He also developed mild ascites treated with low-dose oral furosemide.

The patient was started on sirolimus in addition to his ongoing immunosuppressive therapy in April 2016 for short-term recurrence-free survival of HCC status post-liver transplantation. At a follow-up appointment, he complained of increasing urinary frequency, nocturia, and “frothy” urine. He was also found to have increased proteinuria (1.5 grams). His hepatic function panel at this time revealed rising liver enzymes, which were concerning for possible recurrent NASH or acute rejection. Liver biopsy was performed, which showed steatohepatitis with perisinusoidal fibrosis compatible with NASH (non-alcoholic fatty liver disease (NAFLD) score of 5/8) and no evidence of acute cellular rejection. The sirolimus was stopped in August 2016, and his symptoms subsequently resolved.

Surveillance CT imaging for HCC in setting of the recurrent NASH and history of RCC continued at every three-month interval from February 2016 to February 2020 and then every six months from February 2020 to June 2022, each continuing to demonstrate no evidence of recurrent HCC or RCC despite high immunosuppression therapy. He had significant progression of recurrent NASH from 2016 to 2018 to stage 3 bridging fibrosis complicated by portal hypertension, splenomegaly, and esophageal varices with increased risk of recurrent cirrhosis. In June 2022, it was determined that surveillance could be stopped since it was more than six years since the patient underwent liver transplant and more than seven years post-cryoablation therapy for RCC without evidence of recurrence on appropriate serial surveillance.

Most recent imaging continues to demonstrate a 9-mm enhancing focus (unchanged since 2013) in the pancreatic tail that could represent a small neuroendocrine tumor. The patient is scheduled to undergo CT with pancreas protocol in one year in June 2023.

## Discussion

Extensive work-up during transplant evaluation aims at early detection of any early stage cancer in patients awaiting a solid organ transplant as post-transplant de novo or recurrent cancer is a known complication of immune suppression. Certain cancers have a more aggressive course than others and can be considered as an absolute contraindication especially in late stages as they might have a poor prognosis. With appropriately treated stage 1a RCC, there is no wait time before transplantation, as the five-year recurrence-free survival is 95-98% [7]. However, the standard treatment in these patients before a transplant would be nephrectomy. Occasionally, there could be patients who are discovered to have an SRM and cannot undergo surgical treatment due to organ failure. These significantly high-risk patients could potentially receive a life-saving transplant (heart, liver, lungs) followed by surgical resection a few months post-transplantation.

According to the AUA guidelines, thermal ablation is considered a standard of care for SRMs less than 3 cm [8]. This care report provides evidence that cryoablation is potentially a safe and highly effective means of treating RCC in high-risk patients who are too sick to undergo nephrectomy, such as those with end-stage liver disease and portal hypertension. Supported by long-term follow-up at seven years, cryoablation is an effective and appropriate option for patients undergoing liver transplant evaluation. However, the recurrence rate in these patients after transplant and in the presence of immunosuppression remains unknown.

We described in this case report a stage 1a RCC that was successfully treated with cryoablation before transplant and received no induction immunosuppression and standard triple maintenance post-transplant immunosuppression without recurrence of RCC. This patient is free of recurrence at seven years post-liver transplant without any adjuvant therapy for RCC. It is also notable that he did not receive any reduction in his post-transplant immunosuppression and has continued to remain free of recurrence. This is a first step to showing that cryoablation is potentially a safe and highly effective means of treating RCC in patients who are not candidates for nephrectomy prior to liver transplant.

The strength of this report is our long-term follow-up of seven years and the patient’s compliance with surveillance imaging. Obvious shortcomings of this report include the sample size of one patient. Larger studies are needed to demonstrate long-term recurrence-free survival to be a reproducible outcome and to establish guidelines for which stages of RCC would be candidates for cryoablation alone as well as the optimal duration of surveillance in the setting of lifelong immunosuppression. Due to the rarity of RCC as a finding prior to liver transplant, this poses a significant barrier to performing these further studies. Despite this, we believe that treatment of early stage RCC with cryoablation prior to liver transplant is a viable treatment option if patients are not candidates for traditional surgical resection.

## Conclusions

Cryoablation is potentially a safe and highly effective means of treating RCC in high-risk patients who are not candidates to undergo nephrectomy, such as those with end-stage liver disease and portal hypertension. This case report reveals the potential for clinically significant, extended recurrence-free survival from a potentially aggressive malignancy despite standard triple immunosuppressive therapy after OLT. While there have been a handful of cases documented n RCC prior to OLT, this is the only case that does not manage this extrahepatic malignancy with radical or partial nephrectomy. Considering no recurrence at seven years, this outcome opens the door to study the use of less invasive therapies such as percutaneous cryoablation to successfully treat malignancies in immunosuppressed patients or patients subject to medical immunosuppression after solid organ transplantation.
